# Pathogenic and Low-Frequency Variants in Children With Central Precocious Puberty

**DOI:** 10.3389/fendo.2021.745048

**Published:** 2021-09-24

**Authors:** Vassos Neocleous, Pavlos Fanis, Meropi Toumba, Barbara Gorka, Ioanna Kousiappa, George A. Tanteles, Michalis Iasonides, Nicolas C. Nicolaides, Yiolanda P. Christou, Kyriaki Michailidou, Stella Nicolaou, Savvas S. Papacostas, Athanasios Christoforidis, Andreas Kyriakou, Dimitrios Vlachakis, Nicos Skordis, Leonidas A. Phylactou

**Affiliations:** ^1^ Department of Molecular Genetics, Function and Therapy, The Cyprus Institute of Neurology and Genetics, Nicosia, Cyprus; ^2^ Cyprus School of Molecular Medicine, Nicosia, Cyprus; ^3^ Child Endocrine Care, Department of Pediatrics, Aretaeio Hospital, Nicosia, Cyprus; ^4^ Department of Neurobiology, The Cyprus Institute of Neurology and Genetics, Nicosia, Cyprus; ^5^ Department of Clinical Genetics, The Cyprus Institute of Neurology and Genetics, Nicosia, Cyprus; ^6^ Department of Pediatrics, Iliaktida Paediatric & Adolescent Medical Centre, Limassol, Cyprus; ^7^ University of Nicosia Medical School, Nicosia, Cyprus; ^8^ Division of Endocrinology, Diabetes and Metabolism, First Department of Pediatrics, National and Kapodistrian University of Athens Medical School, “Aghia Sophia” Children’s Hospital, Athens, Greece; ^9^ Division of Endocrinology and Metabolism, Biomedical Research Foundation of the Academy of Athens, Athens, Greece; ^10^ Biostatistics Unit, The Cyprus Institute of Neurology and Genetics, Nicosia, Cyprus; ^11^ Division of Pediatric Endocrinology, Archbishop Makarios III Hospital, Nicosia, Cyprus; ^12^ Centre for Neuroscience and Integrative Brain Research (CENIBRE), University of Nicosia, Nicosia, Cyprus; ^13^ First Pediatric Department, School of Medicine, Faculty of Medical Sciences, Aristotle University of Thessaloniki, Thessaloniki, Greece; ^14^ Developmental Endocrinology Research Group, School of Medicine, University of Glasgow, Glasgow, United Kingdom; ^15^ Laboratory of Genetics, Department of Biotechnology, School of Applied Biology and Biotechnology, Agricultural University of Athens, Athens, Greece; ^16^ Lab of Molecular Endocrinology, Center of Clinical, Experimental Surgery and Translational Research, Biomedical Research Foundation of the Academy of Athens, Athens, Greece; ^17^ Department of Informatics, Faculty of Natural and Mathematical Sciences, King’s College London, London, United Kingdom; ^18^ St George’s, University of London Medical School, University of Nicosia, Nicosia, Cyprus; ^19^ Division of Pediatric Endocrinology, Paedi Center for specialized Pediatrics, Nicosia, Cyprus

**Keywords:** central precocious puberty, *DLK1*, *KISS1*, *KISS1R, MAGEL2*, next-generation sequencing, *MKRN3*

## Abstract

**Background:**

Central precocious puberty (CPP) due to premature activation of GnRH secretion results in early epiphyseal fusion and to a significant compromise in the achieved final adult height. Currently, few genetic determinants of children with CPP have been described. In this translational study, rare sequence variants in *MKRN3*, *DLK1*, *KISS1*, and *KISS1R* genes were investigated in patients with CPP.

**Methods:**

Fifty-four index girls and two index boys with CPP were first tested by Sanger sequencing for the *MKRN3* gene. All children found negative (*n* = 44) for the *MKRN3* gene were further investigated by whole exome sequencing (WES). In the latter analysis, the status of variants in genes known to be related with pubertal timing was compared with an in-house Cypriot control cohort (n = 43). The identified rare variants were initially examined by *in silico* computational algorithms and confirmed by Sanger sequencing. Additionally, a genetic network for the *MKRN3* gene, mimicking a holistic regulatory depiction of the crosstalk between *MKRN3* and other genes was designed.

**Results:**

Three previously described pathogenic *MKRN3* variants located in the coding region of the gene were identified in 12 index girls with CPP. The most prevalent pathogenic *MKRN3* variant p.Gly312Asp was exclusively found among the Cypriot CPP cohort, indicating a founder effect phenomenon. Seven other CPP girls harbored rare likely pathogenic upstream variants in the *MKRN3.* Among the 44 CPP patients submitted to WES, nine rare *DLK1* variants were identified in 11 girls, two rare *KISS1* variants in six girls, and two rare *MAGEL2* variants in five girls. Interestingly, the frequent variant rs10407968 (p.Gly8Ter) of the *KISS1R* gene appeared to be less frequent in the cohort of patients with CPP.

**Conclusion:**

The results of the present study confirm the importance of the MKRN3-imprinted gene in genetics of CPP and its key role in pubertal timing. Overall, the results of the present study have emphasized the importance of an approach that aligns genetics and clinical aspects, which is necessary for the management and treatment of CPP.

## Introduction

Central Precocious Puberty (CPP) results from the premature activation of the hypothalamic-pituitary-gonadal (HPG) axis. It is clinically defined by the development of progressive secondary sexual characteristics before the age of 8 years in girls and 9 years in boys; mainly breast development in girls and testicular enlargement in boys, acceleration in linear growth and pubertal mood changes (1, 2). CPP has been reported to represent 80% of patients with precocious puberty and to be predominant in girls (2–4). The benchmark for the establishment of a hormonal diagnosis includes the evaluation of predominant luteinizing hormone (LH) over follicle-stimulating hormone (FSH) levels after the administration with exogenous luteinizing hormone-releasing hormone (LHRH) agonists (5) or/and elevated estradiol in girls. In boys, elevated testosterone is an additional hormonal criterion. Nevertheless, basal LH levels are also being used in some settings, as upgraded laboratory methodologies for LH assays have become accessible (6). Additionally, in patients with CPP, hand X-rays can show bone age advancement and pelvic ultrasound in girls can confirm the progression of ovarian function and increased uterus size (7).

Family history of CPP has been recognized in up to 27.5% of cases with an autosomal mode of inheritance. Few genes have been described as causative of CPP, involving both excitatory and inhibitory pathways of GnRH secretion (8–10). At this time, genetic aberrations associated with the *Makorin Ring Finger Protein 3 (MKRN3)* gene are the leading genetic etiology of CPP (9). Since the groundbreaking discovery of loss-of-function mutations in the *MKRN3* gene (11), numerous other studies followed and reported more than 40 novel variants, including missense, nonsense, and frameshift mutations in MKRN3 across families with CPP in a broad spectrum of geographical regions (9, 12). *MKRN3* gene is located in the Prader-Willi syndrome (PWS)-related region (15q11-q13) on chromosome 15. The maternal allele of the gene is imprinted therefore is expressed only from the paternal allele. All affected patients reported with familial CPP inherited the *MKRN3* mutations from their fathers (9, 13–28). MKRN3 is composed of five zinc-finger domains: three C3H1 motifs, one C3HC4 RING motif, and one MKRN-specific Cys-His domain (29). C3H1 zinc-finger motifs are responsible for RNA binding while the RING motif is detected in E3 ubiquitin ligases and hereafter, it is anticipated that it has an ubiquitin-ligase activity (30). Recently, it has been demonstrated that auto-ubiquitination of the MKRN3 protein is diminished as a result of mutations located on the C3HC4 RING motif (31). Additional work by Li et al. (32) demonstrated that MKRN3 epigenetically regulates the transcription of *GNRH1* through ubiquitination of MBD3 and controls the onset of mammalian puberty.

The MKRN3 is highly expressed in the arcuate nucleus of the developing brain and is believed to be involved in protein degradation leading in that way to an inhibitory effect of the pulsatile GnRH secretion (30). The exact mechanism that accomplish this effect or by which mechanism MKRN3 deficiency results in early reactivation of GnRH secretion is still under investigation. Although, the majority of the reported studies refer to loss-of-function mutations in the coding region of *MKRN3*, defects in the regulatory regions of the gene were recently described in three studies (23, 33, 34). In the first study, a four nucleotide deletion (c.-150_-147delTCAG) in the proximal promoter region of the *MKRN3* gene was found to be responsible for causing CPP (23). In the second study, a single nucleotide substitution at position 19 (*MKRN3:g.+19C>T*) from the transcription start site (TSS) in the 5′-UTR region of the *MKRN3* gene was also associated with CPP (33). Finally, in a recent third study by our group, we report three novel heterozygous mutations located in the proximal promoter and one in the 5′-UTR region of the *MKRN3* gene in a total number of seven nonrelated girls with CPP. Four girls carried the *MKRN3:g.-865G>A* mutation, one the *MKRN3:g.-166G>A*, and one the *MKRN3:g.-886C>T* mutation; all variants were located in the proximal promoter of the gene. Interestingly, a 7.6-year-old girl with CPP was identified with the novel *MKRN3:g.+13C>T* mutation in the 5′-UTR region (34).

To date, only two gain-of-function mutations in the *KISS1/KISS1R* pathway have been reported: a girl with *KISS1R* missense mutation and a boy with *KISS1* missense mutation. Their presence caused upregulation of the KISS1/KISS1R system leading to GnRH secretion and HPG activation (9, 35, 36). Subsequently, several studies did not identify gain-of-function *KISS1* and *KISS1R* mutations in cohorts of children with CPP, suggesting these may be extremely rare causes of the disorder (37–39). Lately, several mutations in a second maternally imprinted and paternally expressed gene, the Delta-like noncanonical Notch ligand 1 (*DLK1*), have also been associated with CPP (9). *DLK1* is also known as preadipocyte factor 1 (*Pref-1*) and is involved in the Notch signaling pathway as an adipocyte modulator (40). Studies identified loss-of-function mutations in the *DLK1* gene (deletions and frameshifts) as a rare cause of CPP (41–43), strengthening a significant role of this factor in human pubertal timing and the age of menarche (44).

In the present study, we have assembled a cohort of patients with CPP to investigate genetic determinants implicated in the disorder, based on the recent global literature data. By using an expanded GnRH/CPP-associated gene panel, we examined the involvement of such genes.

## Materials and Methods

### Patients

Fifty-four index girls (96.4%) and two (3.6%) index boys with CPP were referred for genetic investigation to the Department of Molecular Genetics, Function and Therapy at the Cyprus Institute of Neurology and Genetics. All children included in the study fulfilled the criteria for CPP diagnosis. Girls presented with breast development Tanner stage 2 before the age of 8 years and boys presented with testicular enlargement more than 4 ml in volume measured with Prader orchidometer before the age of 9 years. Elevated basal or stimulated gonadotrophins with LH predominance confirmed the diagnosis. Elevated estradiol levels in girls or testosterone levels in boys and imaging studies; bone age X-ray evaluated by Greulich and Pyle method (45), pelvic ultrasound, and hypothalamus-pituitary MRI with contrast were used as additional diagnostic tools.

At a first stage, all index patients with CPP were tested by Sanger sequencing using appropriately designed primers for the *MKRN3* (RefSeq NM_005664.4) gene. Further testing by whole exome sequencing (WES) was performed on the negative *MKRN3-*tested patients. Written informed consent was obtained from parents of all patients under the age of 16 that participated in the study. The project was approved by the Cyprus National Ethics Committee, and all methods were performed in accordance with the relevant guidelines and regulations.

### Genetic Analysis

Genomic DNA was extracted from peripheral blood using the Gentra Puregene Kit (Qiagen, Valencia, CA, USA) according to the manufacturer’s instructions. The DNA purity was measured using the Nanodrop ND-1000 spectrophotometer (NanoDrop Technologies, Wilmington, DE, USA). Prior to library preparation for whole exome sequencing (WES), genomic DNA was quantified using the Qubit dsDNA BR Assay Kit (Invitrogen, Life Technologies, Eugene, OR, USA) on a Qubit^®^ 2.0 Fluorometer (Invitrogen, Life Technologies, Eugene, OR, USA). WES was performed by using the TruSeq Exome Kit (Illumina Inc., San Diego, CA, USA) with paired-end 150-bp reads. NGS was performed using the NextSeq 500/550 High Output Kit v2.5 (150 cycles) on a NextSeq500 system (Illumina Inc., San Diego, CA, USA). The FastQC quality control tool (http://www.bioinformatics.babraham.ac.uk/projects/fastqc/) was used to evaluate the quality of the WES procedure. The mean target coverage of the whole exome was 62.13×. Specifically, 10× coverage was reached for 92.34% of the nucleotides, 20× coverage for 86.03% of the nucleotides and 30× coverage for 76.96% of the nucleotides, indicating that the WES reaction was of sufficiently high quality for subsequent analysis.

### Variant Analysis

The fastq data obtained by WES were processed using an in-house bioinformatics pipeline. Briefly, all variants were inputted into the VarApp Browser, filtered, and aligned to the human reference genome GRCh38.p12, hg38 assembly. VarApp is a graphical user interface, which supports GEMINI (18). Variants in genes previously associated with pubertal onset and precocious puberty were further analyzed using the Qualimap v2.2.1 tool (46) to calculate the target coverage. Mean target coverage was 60× of the selected genes (**Supplementary Table S1**). Variants in these genes were additionally filtered using the VarApp Browser for minor allele frequencies of less than 1% in public databases such as 1,000 genomes, ExAC browser, and Exome Sequencing Project (ESP). Moreover, variants were filtered and selected according to their impact such as frameshift, splice acceptor, splice donor, start lost, stop gained, stop lost, inframe deletion, inframe insertion, missense, protein altering, and splice region. In addition, variants were filtered by the VarApp Browser for their pathogenicity by two *in silico* tools, SIFT and Polyphen2. Population-specific data from an in-house WES library composed of 43 randomly selected samples of Cypriot origin were used as an in-house control cohort. All variants identified were confirmed by Sanger sequencing. Finally, the variants were categorized for their pathogenicity using the standards and guidelines of the American College of Medical Genetics and Genomics and the Association for Molecular Pathology (47). Any additional variants identified in other genes resulting from the more in-depth WES analysis were also analyzed using the described above methodology.

### Genetic Network Modeling

Finally, the genetic network was constructed using the genemania platform (https://genemania.org). *MKRN3* was used as the anchor gene and a full association network was designed including all genes that crosstalk with *MKRN3*. Namely, at the time of the analysis genemania database was indexing 2,830 association networks containing 660,554,667 interactions mapped on 16,6691 genes. For the purposes of this study, only human genes were studied for protein and genetic interactions, similar protein domains, genetic and biochemical pathways, colocalization, and coexpression of genes.

## Results

### Clinical Findings

The more prevalent clinical, biochemical, and imaging features regarding the cohort of 56 index patients with CPP (54 girls and two boys) are summarized in **Table 1**. All patients fulfilled the clinical criteria of central precocious puberty; breast development in girls before the age of 8 years and testicular enlargement of more than 4 ml in boys before the age of 9 years. The majority of the girls presented at Tanner stage 2 of breast development. Only two girls presented with premature menarche both at age 9.5 years. The majority of the girls underwent LHRH test which showed a predominance of the LH over FSH levels. Bone age advancement was above 2 years compared with the chronological age in the majority of the patients. MRI of the hypothalamus-pituitary was reported as normal in all the patients included in the study. Twelve of the patients confirmed positive family history of CPP in either parental side.

**Table 1:** Clinical and hormonal features of 56 patients (*n* = 54 girls; *n* = 2 boys) with CPP.

	Mean ± SD
Age of onset for the 54 girls (years)	6.71 ± 0.96
Age of onset for the 2 boys (years)	8.05
Age of presentation for the 54 girls (years)	7.52 ± 1.28
Age of presentation for the 2 boys (years)	9.20
Bone age advancement (years)	2.55 ± 1.60
Height at presentation SDS	0.92 ± 0.85
Weight at presentation SDS	0.86 ± 0.89
Midparental target height SDS	−0.41 ± 0.76
Basal LH (IU/L)	2.11 ± 1.82
LH peak (IU/L)	15.3 ± 15.63
Basal FSH (IU/L)	4.33 ± 2.45
FSH peak (IU/L)	12.74 ± 6.90
Estradiol (pg/ml) (prepubertal level <20)	39.76 ± 19.86
Duration of tx (years)	2.13 ± 1.8
Height SDS at the end of tx	0.78 ± 0.71
BMI SDS at the end of tx	0.61 ± 1.12
Age of menarche after the end of tx (years)	12.12 ± 1.23

### Pathogenic Genetic Defects in *MKRN3* Gene

**Figure 1:**
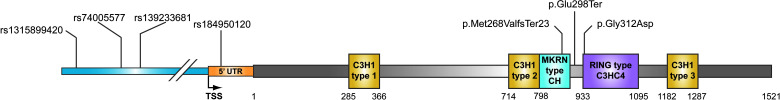
Schematic representation of the *MKRN3* gene with the mutations/variations identified in this study. The five zinc-finger domains in the coding region of *MKRN3* gene are indicated: three C3H1 motifs, one C3HC4 RING motif, and one MKRN-specific Cys-His domain. MKRN3 domains are positioned in relation to start codon *via* nucleotide numbering of coding region.

In 12 index girls with CPP, we have also identified three different mutations in the coding region of the *MKRN3* gene. More specifically, the missense p.Gly312Asp was identified in seven index girls, the frameshift p.Met268ValfsTer23 in four index girls, and the nonsense p.Glu298Ter in one index girl with CPP (**Table 2**). Segregation analysis of all 12 index girls with *MKRN3* mutations identified paternal inheritance. Family history of early menarche was reported in nine of 12 paternal grandmothers of the CPP girls identified with *MKRN3* gene mutations. The upstream variants of uncertain significance (VUS) with evidence for likely pathogenic g.23564798C>T (rs74005577) and g.23564819G>A (rs139233681) were respectively reported in two and four CPP index girls, and DNA sequencing of both parents revealed these variants only in the fathers. The low-frequency VUS (0.000016, TOPMED) g.23565172G>A (rs1315899420) 2KB upstream variant was also detected in one female CPP index patient. Finally, the likely pathogenic 5′-UTR g.23565172G>A (rs184950120) was detected in a CPP index girl and segregation analysis also identified paternal inheritance (**Figure 1** and **Table 2**). The clinical and hormonal findings of the above eight CPP girls identified with the variants in the noncoding regions of the *MKRN3* gene are summarized in **Table 3**.

**Table 2:** *MKRN3* pathogenic and likely pathogenic variants identified in 20 non related females with CPP.

CPP patients	Gene	Refseq (GRCh38.p12)	Variant identified (rs No.)	Protein	Variant classification	Previously described
7	*MKRN3*	NM_005664.4:c.935G>A	–	p.Gly312Asp	Pathogenic	(14)
4	*MKRN3*	NM_005664.4:c.802_803del	–	p.Met268ValfsTer23	Pathogenic	(25)
1	*MKRN3*	NM_005664.4:c892G>T	–	p.Glu298Ter	Pathogenic	(16)
2	*MKRN3*	NC_000015.10:g.23564798C>T	rs74005577	Upstream variant	Variant of uncertain significance (VUS) with evidence for likely pathogenic	(34)
4	*MKRN3*	NC_000015.10:g.23564819G>A	rs139233681	Upstream variant	Variant of uncertain significance (VUS) with evidence for likely pathogenic	(34)
1	*MKRN3*	NM_005664.4:c.-87C>T	rs184950120	5′-UTR variant	Likely pathogenic	(34)
1	*MKRN3*	NC_000015.10:g.23565172G>A	rs1315899420	Upstream variant	Variant of uncertain significance (VUS)	

**Table 3:** Clinical and hormonal findings of CPP girls identified with variants in the noncoding regions of *MKRN3* gene.

CPP patient	*MKRN3* promoter variant (rs No.)	Age of onset (years)	Age of presentation (years)	Height (cm)	Height SD	Weight SD	Weight (kg)	Tanner stage*	Basal LH (U/L)	LH 30 min (U/L)	LH 60 min (U/L)	Basal FSH (U/L)	FSH 30 min (U/L)	FSH 60 min (U/L)	Estradiol (pg/ml)	Bone age	Pelvic ultrasound^b^	MRI (brain-pituitary)	Other conditions	Age of menarche (years)
1	rs139233681 (G>A)	7	7.8	N/A	N/A	N/A	N/A	B4P3A1	2.4	19	21	3.2	14	12	–	12	Pubertal	Normal	–	–
2	rs139233681 (G>A)	7.38	7.38	119	(−)0.88	(−)0.18	20	B2P1A2	1.95	8.9	7.7	3.5	9	7	60.2	10	Pubertal	Normal	Hearing loss (cochlear implants)	11.9
3	rs139233681 (G>A)	N/A	9.5	N/A	N/A	N/A	N/A	B5P4A2	4.8	–	–	5.1	–	–	–	11.5	Pubertal	–	–	Age at presentation (9.5 years) with menarche
4	rs139233681 (G>A)	8	8.2	132	0.53	0.54	26.5	B2P2A2	–	–	–	–	–	–	–	N/A	–	–	–	–
5	rs184950120 (C>T)	7.6	9.2	142	1.32	2.55	55	B3P2A2	4.6	–	–	7.13	–	–	38.8	11.3	Pubertal	–	Obesity—heterozygous for MC4R:p.Lys71Asn mutation	9.5
6	rs74005577 (C>T)	N/A	8.3	N/A	N/A	N/A	N/A	B4P4A4	3	12	9	3	5	4	–	N/A	–	–	–	–
7	rs74005577 (C>T)	7.5	9.5	144	1.39	2.1	48	B5P5A5	–	–	–	–	–	–	–	N/A	–	–	–	–
8	rs1315899420 (G>A)	5.7	6.7	127	1.42	0.77	25	B2P1A2	0.5	10.2	11.4	2.8	9.4	12.7	16	8.5	Pubertal	Microadenoma 2 mm	–	–

*Tanner stage. B, breast development; P, pubic hair; A, under arm hair. ^b^Pelvic ultrasound: pubertal determined as with uterus size >3 cm and ovarian volume >2.5 ml with the presence of multiple follicles. NA, Not Available.

### Other Genetic Findings in the Cohort of CPP Patients

Forty-four out of the total 56 index patients with CPP that were initially tested negative for *MKRN3* mutations have also been submitted to WES. Among the 44 CPP patients submitted to WES, nine rare *DLK1* variants were identified in 11 girls and two rare *KISS1* variants in six girls. Interestingly, the frequent variant rs10407968 (p.Gly8Ter) of the *KISS1R* gene appeared to be less frequent in the cohort of patients with CPP vs. the cohort of age-matched controls (Chi-square or Fisher’s exact test = 0.011753) (**Supplementary Table S2**).

Additionally, in the cohort of CPP patients under investigation, a number of other more frequent variants in a series of genes (*KISS1R*, *TAC3*, *GNRH1*, *GNRHR*, *LHCGR*, *MAGEL2*, and *FSHR*) also directly or indirectly involved in pubertal control and activation have been observed (**Supplementary Table S2**). The more in-depth WES analysis did not identify any variants in other candidate genes.

### Genetic Network Modeling for the *MKRN3* Gene

**Figure 2:**
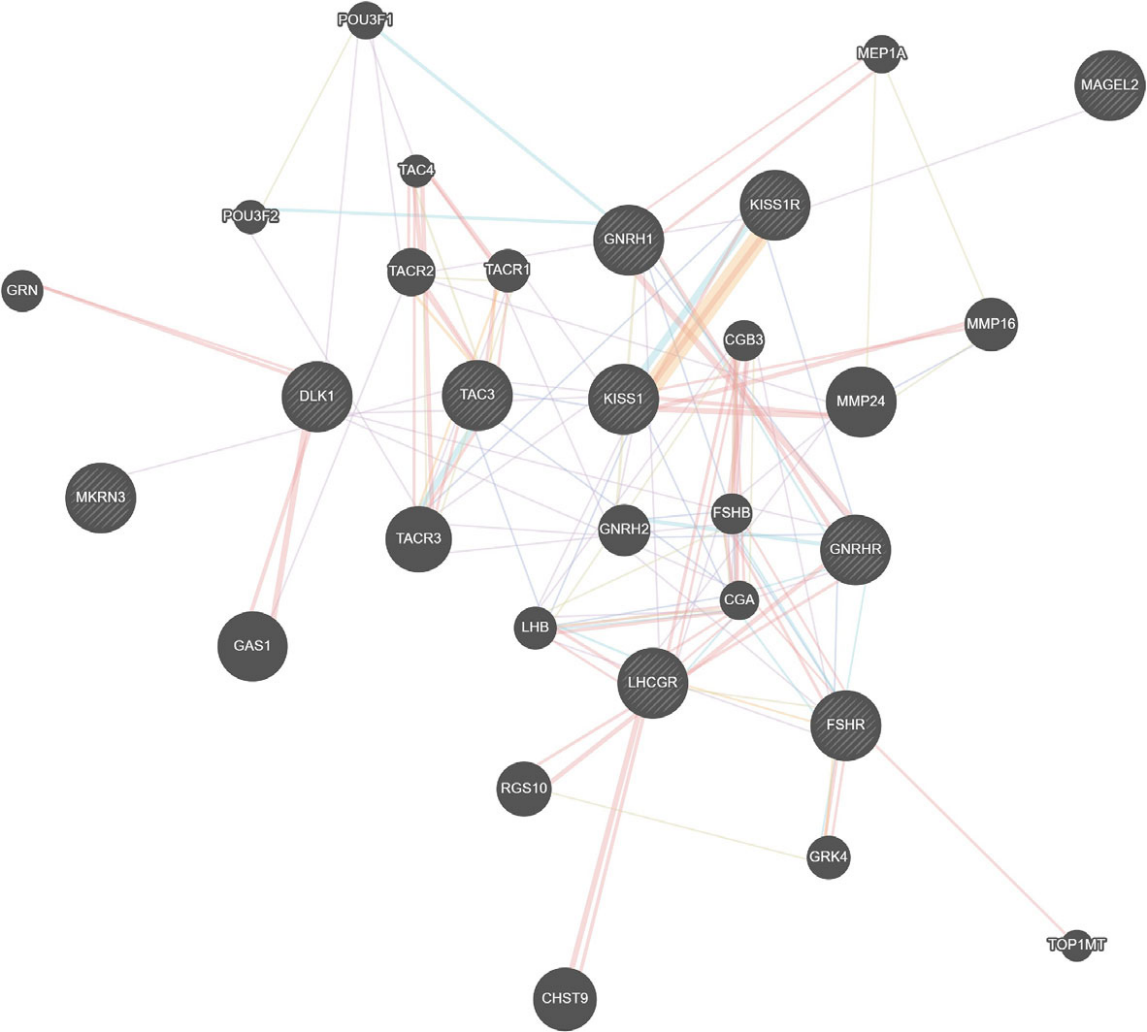
Genetic network modeling for the *MKRN3* gene in a holistic regulatory depiction of the crosstalk between *MKRN3* and other genes.

The genetic network designed for the *MKRN3* gene is a holistic regulatory depiction of the crosstalk between *MKRN3* and other genes (**Figure 2**). Notably, *MKRN3* directly interplays with *TAC3*, while it is only one node away (*TAC3*) from *KISS1* and *KISS1R*. *TAC3* is a gene that encodes for a member of the tachykinin family of secreted neuropeptides. The mature peptide is the result of the proteolytically processed fully encoded preproprotein and is primarily expressed in the central and peripheral nervous systems. *TAC3* has a role of a neurotransmitter, and it is well established that *TAC3* mutations can lead to normosmic hypogonadotropic hypogonadism. Likewise, the *KISS1* and *KISS1R* genes are linked mainly not only to cancer as they act as metastasis suppressor genes (mainly suppressing melanomas and breast carcinoma metastases) but also to central precocious puberty as kisspeptin (protein product of these genes) regulates the pubertal activation of GnRH neurons by stimulating gonadotropin-releasing hormone (GnRH)-induced gonadotropin secretion.

## Discussion

The present study investigated the genetic impact in patients with CPP by using conventional sequencing and high-throughput whole exome sequencing. The study included 56 unrelated children (54 girls and two boys) with CPP presenting with a variable clinical picture at presentation regarding the stage of puberty (**Table 1**). The majority presented with thelarche at Tanner stage 2 of puberty before the age of 8 years. However, two of them presented with premature menarche and Tanner stage 5 of puberty before the age of 9.5 years. The two boys presented with testicular enlargement (testicular size of more than 5 ml) and Tanner stage 2 of puberty before the age of 9 years. Results from the present, in conjunction with previous studies from our group (14, 34), indicated that pathogenic (**Table 2**) or variants of uncertain significance (VUS) (**Table 3**) in the *MKRN3* gene are the most prevalent cause of CPP in our cohort of Cypriot patients, in line with the current published literature (12). The *MKRN3* p.Gly312Asp pathogenic variant, first reported in 2016 by Neocleous et al. (14), was the most predominant genetic defect (seven cases) of the present study, followed by the previously reported p.Met268ValfsTer23 pathogenic variant identified in four cases (25) (**Table 2**). The two previously reported by Fanis et al. (34) heterozygous VUS with evidence of likely pathogenic activity g.23564798C>T (rs74005577) and g.23564819G>A (rs139233681) and the VUS never described before to be associated with CPP g.23565272G>A (rs1315899420) were identified in seven nonrelated girls with CPP (**Table 2**). All three variants, rs74005577, rs139233681, and rs1315899420 are respectively located −886, −865, and −512 nt upstream to the transcription site in the proximal promoter region of the *MKRN3* gene. In the same study by Fanis et al. (34), a 5′-UTR (+13 nt downstream to the transcription start site) novel mutation was also identified in a CPP girl. Most of the reported studies describe loss-of-function pathogenic variants in the coding region of the *MKRN3* gene, and to date, only a couple of studies reported pathogenic variants linked to CPP outside the coding region of the gene. These studies include a recent study that described a single nucleotide substitution at position 19 from the transcription start site (TSS) in the 5′-UTR region of the *MKRN3* and another one that reported four nucleotide deletions (c.-150_-147delTCAG) in the proximal promoter region of the gene (23, 33).

In the last two decades, studies have reported monogenic causes of CPP, including *MKRN3* loss-of-function mutations as the most common (11, 35, 36, 43). In the present study, all patients that were tested negative for pathogenic variants in the *MKRN3* gene were further investigated by whole exome NGS analysis. We identified numerous single nucleotide variants (SNVs) in various genes known to be involved in pubertal control. Seven SNVs in the *DLK1* gene in eight different female CPP patients of our cohort were observed (**Supplementary Table S2**). Rare pathogenic variants in the *DLK1* gene have recently been reported as an infrequent cause of CPP (41–43), therefore such a connection of the rare SNVs observed in the cases of our cohort as possible associated factors could not be excluded. Up-to-date, *DLK1* along with *MKRN3* are two of the four known monogenic causes of CPP and that are both imprinted. This elevates the possibility of imprinting to play a significant role in the regulation of puberty and that other imprinted genes might also be involved in the pubertal control (9, 48).

Interestingly, the previously reported single nucleotide polymorphism (SNP) rs10407968 (p.Gly8=) in *KISS1R* gene (49) was detected with a minor allelic frequency (MAF) of 8.33 in the CPP cohort of patients of the present study. A notable and statistically significant difference was observed for the *KISS1R* rs10407968 between the CPP cohort of patients of the present study (MAF = 8.33) vs. the Cypriot control subjects (MAF = 23.25). An assumption that can be said with observing the less-frequent rs10407968 MAF in CPP patients is that it could be behaving as a reducing agent for the risk of CPP. The above assumption is primarily made by the fact that activating mutations were reported in the G protein-coupled receptor (*KISS1R*) and its ligand (*KISS1*) genes as causes of early GnRH secretion in patients with CPP (35, 36). Additional investigation is needed to examine this possibility and the potential functional effects of the rs10407968 SNP. The genetic network modeling indicated a possible interplay between those genes (**Figure 2**). However, the roles of the remaining genes that have been identified by the clustering algorithm merit to be further investigated, as they may provide invaluable insights as regulatory elements of the genetic, epigenetic, and developmental traits of central precocious puberty, as well as putative novel pharmacological targets under the prism of personalized and precision medicine.

In the present study, we confirmed the key role of the imprinted *MKRN3* gene as the most common cause of monogenic CPP in the Cyprus cohort. The newly described p.Gly312Asp missense loss-of-function pathogenic variant was identified as the most prevalent among the tested Cypriot CPP cohort. This could be most likely due to the founder effect, a frequent phenomenon in the Cypriot population (50–54). Additionally, other findings of the present study indicate that causing variants can also exist in the *MKRN3* proximal promoter and 5′-UTR region and which can also be considered contributing factors to CPP. Overall, the results of the present study have emphasized the importance of an approach that aligns genetics and clinical aspects.

## Data Availability Statement

The datasets presented in this study can be found in online repositories. The names of the repository/repositories and accession number(s) can be found below: (https://datadryad.org/stash), DOI (https://doi.org/10.5061/dryad.8pk0p2nnj).

## Ethics Statement

The studies involving human participants were reviewed and approved by Cyprus National Ethics Committee. Written informed consent to participate in this study was provided by the participants’ legal guardian/next of kin.

## Author Contributions

VN, PF, MT, NS, and LP contributed to the conception, design, and interpretation of the project. BG, IK, GT, MI, YC, KM, SN, SP, AC, AK, and DV contributed to the experimental part and data analysis of the project. MT, NN, AK, and NS contributed to drafting or revising intellectual content of the manuscript. VN, PF, MT NS, and LP had primary responsibility for final content. All authors contributed to the article and approved the submitted version.

## Funding

This study received funding from A.G Leventis Foundation & RCB Bank Ltd. The funders were not involved in the study design, collection, analysis, interpretation of data, the writing of this article or the decision to submit it for publication.

## Conflict of Interest

The authors declare that the research was conducted in the absence of any commercial or financial relationships that could be construed as a potential conflict of interest.

## Publisher’s Note

All claims expressed in this article are solely those of the authors and do not necessarily represent those of their affiliated organizations, or those of the publisher, the editors and the reviewers. Any product that may be evaluated in this article, or claim that may be made by its manufacturer, is not guaranteed or endorsed by the publisher.
