# Supplementary material for: Pathogenic and Low-Frequency Variants in Children With Central Precocious Puberty
Source: Front Endocrinol (Lausanne). 2021 Sep 24;12:745048. doi: 10.3389/fendo.2021.745048 (PMC8498594; doi:10.3389/fendo.2021.745048)
Supplement: Supplementary file 1 [file Table_1.docx]

**Supplementary table 1.** Genes involved in pubertal onset and were selected for variant analysis. For each gene the corresponding genetic information is indicated. The mean target coverage of the target region for each gene obtained by WES is designated in a separate column.

| **Gene** | **Transcript ID** | **Reference Sequence (RefSeq)** | **Mean target coverage** |
| --- | --- | --- | --- |
| *MKRN3* | ENST00000314520.6 | NM_005664 | 46.86X |
| *DLK1* | ENST00000341267.9 | NM_001317172 | 12.79X |
| *KISS1* | ENST00000367194.5 | NM_002256 | 72.98X |
| *KISS1R* | ENST00000234371.10 | NM_032551 | 44.30X |
| *TAC3* | ENST00000393867.5 | NM_013251 | 97.11X |
| *GNRH1* | ENST00000421054.7 | NM_001083111 | 78.83X |
| *GNRHR* | ENST00000226413.5 | NM_000406 | 28.81X |
| *LHCGR* | ENST00000294954.12 | NM_000233 | 86.57X |
| *FSHR* | ENST00000406846.7 | NM_000145 | 98.44X |
| *MAGEL2* | ENST00000650528.1 | NM_019066 | 33.24X |
