# Supplementary material for: Pathogenic and Low-Frequency Variants in Children With Central Precocious Puberty
Source: Front Endocrinol (Lausanne). 2021 Sep 24;12:745048. doi: 10.3389/fendo.2021.745048 (PMC8498594; doi:10.3389/fendo.2021.745048)
Supplement: Supplementary file 2 [file Table_2.docx]

**Supplementary table 2.** NGS analysis identified numerous gene variants in 36 non-related CPP patients that were first tested negative for pathogenic variants in the *MKNR3* gene. The cohort of CPP patients consisted of 35 females and 1 male. The NGS results of the CPP patients were compared with the ones obtained from a Cypriot cohort of controls.

| **Gene** | **Refseq**  **(GRCh38.p12)** | **Variant identified**  **(rs #)** | **Variant identified**  **(Protein)** | **Allele count in present study** | **Number of Homozygotes** | **MAF of present CPP cohort (%)**  **(n=36; 35F, 1M)** | **MAF of Cypriot control samples (%)**  **n=43 (22F, 21M)** | **Chi-square or Fisher’s exact test** | **MAF (%) – gnomAD v2.1.1** | **Previously described** |
| --- | --- | --- | --- | --- | --- | --- | --- | --- | --- | --- |
| *MKRN3* | NM_005664.4:c.663C>T | rs2239669 | p.Pro221= | 20 | 3 | 20/72= 27.78 | 20/86=23.26 | 0.4238 | 27.59 | ([1](#_ENREF_1)) |
| *DLK1* | NM_003836.7:c.779G>A | rs1058009 | p.Ser260Asn | 2 | - | 2/72 = 2.78 | 6/86=6.97 | 0.2923 | 7.01 |  |
| *DLK1* | NM_003836.7:c.564T>C | rs1802710 | p.Ile188Ile | 26 | 6 | 26/72 = 36.11 | 35/86=40.7 | 0.3478 | 63.1 | ([2](#_ENREF_2)) |
| *DLK1* | NM_003836.7:c.99A>G | rs13329039 | p.Gln33Gln | 1 | - | 1/72 = 1.39 | 0/86=0 | N/A | 0.07743 |  |
| *DLK1* | NM_003836.7:c.262+119C>T | rs117667438 | Intron Variant | 2 | - | 2/72 = 2.78 | 0/86=0 | N/A | 0.721 |  |
| *DLK1* | NM_003836.7:c.310G>A | rs2273607 | p.Val104Met | 1 | - | 1/72 = 1.39 | 0/86=0 | N/A | 0.656 |  |
| *DLK1* | NM_003836.7:c.963T>C | rs140051660 | p.Thr248Thr | 1 | - | 1/72 = 1.39 | 0/86=0 | N/A | 0.05339 |  |
| *DLK1* | NM_003836.7:c.231C>T | rs746697940 | p.Thr77Thr | 1 | - | 1/72 = 1.39 | 0/86=0 | N/A | 0.00438 |  |
| *DLK1* | NM_003836.7:c.603C>A | rs34429112 | p.Ile201Ile | 1 | - | 1/72 = 1.39 | 0/86=0 | N/A | 0.0829 |  |
| *DLK1* | NM_003836.7:c.262+129= | rs1315957845 | Intron Variant | 1 | - | 1/72 = 1.39 | Not detected | N/A | 0.000607 |  |
| *DLK1* | NM_003836.7:c.699T>C | rs2295660 | p.Cys233Cys | 1 | - | 1/72 = 1.39 | 0/86=0 | N/A | 1.109 | ([3](#_ENREF_3)) |
| *KISS1* | NM_002256.4:c.242C>T | rs4889 | p.Pro81Arg | 22 | 1 | 22/72 = 30.56 | 31/86=36.04 | 0.5301 | 28.74 | ([4](#_ENREF_4), [5](#_ENREF_5)) |
| *KISS1* | NM_002256.4:c.58G>A | rs12998 | p.Glu20Lys | 2 | - | 2/72 = 2.78 | 2/86=2.33 | 1 | 3.257 |  |
| *KISS1* | NM_002256.4:c.417del | rs71745629 | p.Ter139Trpfs | 18 |  | 18/72 = 25.00 | 29/86=33.72 | 0.2324 | 22.79 | ([6](#_ENREF_6)) |
| *KISS1* | NM_002256.4:c.107A>G | rs35431622 | p.Gln36Arg | 4 | - | 4/72 = 5.56 | 4/86=4.65 | 1 | 5.18 |  |
| *MAGEL2* | NM_019066.5:c.3151C>A | rs2233070 | p.Leu1051Ile | 4 | - | 4/72 = 5.56 | 7/86=8.14 | 0.7553 | 3.237 |  |
| *MAGEL2* | NM_019066.5:c.2886C>T | rs2233068 | p.Ser962= | 1 | - | 1/72 = 1.39 | 2/86=2.32 | 1 | 0.508 |  |
| *KISS1R* | NM_032551.5:c.24A>G | rs10407968 | p.Gly8= | 6 | 2 | 6/72 = 8.33 | 20/86=23.25 | **0.011753** | 16.13 | ([7-9](#_ENREF_7)) |
| *KISS1R* | NM_032551.5:c.1091T>A | rs350132 | p.Leu364His | 53 | 22 | 53/72 = 73.61 | 63/86=73.25 | 0.959845 | 79.42 | ([9](#_ENREF_9)) |
| *TAC3* | NM_013251.4:c.292+162A>G | rs733629 | Intron Variant | 5 | - | 5/72 = 6.94 | 6/86=6.98 | 0.993661 | 9.484 | ([10](#_ENREF_10)) |
| *GNRH1* | NM_000825.3:c.126C>T | rs751508393 | p.Ala42= | 1 | - | 1/72 = 1.39 | 0/86=0 | N/A | 0.003206 |  |
| *GNRH1* | NM_000825.3:c.59G>C | rs6185 | p.Trp20Ser | 10 | 1 | 10/72 = 13.89 | 20/86=23.25 | 0.134884 | 22.99 | ([11](#_ENREF_11), [12](#_ENREF_12)) |
| *GNRHR* | NM_000406.3:c.453C>T | rs4986942 | p.Ser151= | 4 | - | 4/72 = 5.56 | 9/86=10.47 | 0.385 | 7.56 | ([11](#_ENREF_11)) |
| *LHCGR* | NM_000233.4:c.872A>G | rs12470652 | p.Asn291Ser | 2 | - | 2/72 = 2.78 | 0/86=0 | N/A | 3.829 | ([13](#_ENREF_13), [14](#_ENREF_14)) |
| *LHCGR* | NM_000233.4:c.935A>G | rs2293275 | p.Asn312Ser | 50 | 18 | 50/72 = 69.44 | 58/86=67.44 | 0.787507 | 60.23 | ([13-15](#_ENREF_13)) |
| *LHCGR* | NM_000233.4:c.1065T>C | rs11125179 | p.Asp355= | 50 | 18 | 50/72 = 69.44 | 58/86=67.44 | 0.787507 | 60.37 |  |
| *LHCGR* | NM_000233.4:c.50_55dup | rs71245621 | p.Leu17_Gln18dup | 25 | 5 | 25/72 = 34.72 | Not detected | N/A | 26.30 |  |
| *FSHR* | NM_000145.4:c.2039G>A | rs6166 | p.Ser680Asn | 36 | 10 | 36/72 = 50.00 | 39/86=45.35 | 0.559832 | 57.45 | ([16](#_ENREF_16), [17](#_ENREF_17)) |
| *FSHR* | NM_000145.4:c.919G>A | rs6165 | p.Ala307Thr | 36 | 10 | 36/72 = 50.00 | 39/86=45.35 | 0.559832 | 48.858 | ([16](#_ENREF_16), [17](#_ENREF_17)) |

**References**

1. Yi BR, Kim HJ, Park HS, Cho YJ, Kim JY, Yee J, et al. Association between MKRN3 and LIN28B polymorphisms and precocious puberty. *BMC genetics* (2018) 19(1):47. Epub 2018/07/29. doi: 10.1186/s12863-018-0658-z. PubMed PMID: 30053798; PubMed Central PMCID: PMC6062980.

2. Yin D, Xie D, De Vos S, Liu G, Miller CW, Black KL, et al. Imprinting status of DLK1 gene in brain tumors and lymphomas. *International journal of oncology* (2004) 24(4):1011-5. Epub 2004/03/11. PubMed PMID: 15010842.

3. Huang J, Zhang X, Zhang M, Zhu JD, Zhang YL, Lin Y, et al. Up-regulation of DLK1 as an imprinted gene could contribute to human hepatocellular carcinoma. *Carcinogenesis* (2007) 28(5):1094-103. Epub 2006/11/23. doi: 10.1093/carcin/bgl215. PubMed PMID: 17114643.

4. Albalawi FS, Daghestani MH, Daghestani MH, Eldali A, Warsy AS. rs4889 polymorphism in KISS1 gene, its effect on polycystic ovary syndrome development and anthropometric and hormonal parameters in Saudi women. *Journal of biomedical science* (2018) 25(1):50. Epub 2018/06/01. doi: 10.1186/s12929-018-0452-2. PubMed PMID: 29848339; PubMed Central PMCID: PMC5975709.

5. Li D, Wu Y, Cheng J, Liu L, Li X, Chen D, et al. Association of Polymorphisms in the Kisspeptin/GPR54 Pathway Genes With Risk of Early Puberty in Chinese Girls. *The Journal of clinical endocrinology and metabolism* (2020) 105(4). Epub 2020/03/12. doi: 10.1210/clinem/dgz229. PubMed PMID: 32160304.

6. Pare-Brunet L, Sebio A, Salazar J, Berenguer-Llergo A, Rio E, Barnadas A, et al. Genetic variations in the VEGF pathway as prognostic factors in metastatic colorectal cancer patients treated with oxaliplatin-based chemotherapy. *The pharmacogenomics journal* (2015) 15(5):397-404. Epub 2015/02/25. doi: 10.1038/tpj.2015.1. PubMed PMID: 25707392.

7. Ghaemi N, Ghahraman M, Noroozi Asl S, Vakili R, Fardi Golyan F, Moghbeli M, et al. Novel DNA variation of GPR54 gene in familial central precocious puberty. *Ital J Pediatr* (2019) 45(1):10. Epub 2019/01/13. doi: 10.1186/s13052-019-0601-6. PubMed PMID: 30635063; PubMed Central PMCID: PMCPMC6329138.

8. Branavan U, Muneeswaran K, Wijesundera WSS, Senanayake A, Chandrasekharan NV, Wijeyaratne CN. Association of Kiss1 and GPR54 Gene Polymorphisms with Polycystic Ovary Syndrome among Sri Lankan Women. *Biomed Res Int* (2019) 2019:6235680. Epub 2019/04/18. doi: 10.1155/2019/6235680. PubMed PMID: 30993114; PubMed Central PMCID: PMCPMC6434290.

9. Oh YJ, Rhie YJ, Nam HK, Kim HR, Lee KH. Genetic Variations of the KISS1R Gene in Korean Girls with Central Precocious Puberty. *J Korean Med Sci* (2017) 32(1):108-14. Epub 2016/12/04. doi: 10.3346/jkms.2017.32.1.108. PubMed PMID: 27914139; PubMed Central PMCID: PMCPMC5143281.

10. Christofolini DM, Mafra FA, Catto MC, Bianco B, Barbosa CP. New candidate genes associated to endometriosis. *Gynecol Endocrinol* (2019) 35(1):62-5. Epub 2018/07/26. doi: 10.1080/09513590.2018.1499090. PubMed PMID: 30044155.

11. Canzian F, Kaaks R, Cox DG, Henderson KD, Henderson BE, Berg C, et al. Genetic polymorphisms of the GNRH1 and GNRHR genes and risk of breast cancer in the National Cancer Institute Breast and Prostate Cancer Cohort Consortium (BPC3). *BMC Cancer* (2009) 9:257. Epub 2009/07/31. doi: 10.1186/1471-2407-9-257. PubMed PMID: 19640273; PubMed Central PMCID: PMCPMC2729775.

12. Valkenburg O, Uitterlinden AG, Piersma D, Hofman A, Themmen AP, de Jong FH, et al. Genetic polymorphisms of GnRH and gonadotrophic hormone receptors affect the phenotype of polycystic ovary syndrome. *Hum Reprod* (2009) 24(8):2014-22. Epub 2009/05/01. doi: 10.1093/humrep/dep113. PubMed PMID: 19403562.

13. Piersma D, Verhoef-Post M, Look MP, Uitterlinden AG, Pols HA, Berns EM, et al. Polymorphic variations in exon 10 of the luteinizing hormone receptor: functional consequences and associations with breast cancer. *Mol Cell Endocrinol* (2007) 276(1-2):63-70. Epub 2007/08/22. doi: 10.1016/j.mce.2007.06.007. PubMed PMID: 17709176.

14. Haasl RJ, Ahmadi MR, Meethal SV, Gleason CE, Johnson SC, Asthana S, et al. A luteinizing hormone receptor intronic variant is significantly associated with decreased risk of Alzheimer's disease in males carrying an apolipoprotein E epsilon4 allele. *BMC Med Genet* (2008) 9:37. Epub 2008/04/29. doi: 10.1186/1471-2350-9-37. PubMed PMID: 18439297; PubMed Central PMCID: PMCPMC2396156.

15. Simoni M, Tuttelmann F, Michel C, Bockenfeld Y, Nieschlag E, Gromoll J. Polymorphisms of the luteinizing hormone/chorionic gonadotropin receptor gene: association with maldescended testes and male infertility. *Pharmacogenet Genomics* (2008) 18(3):193-200. Epub 2008/02/28. doi: 10.1097/FPC.0b013e3282f4e98c. PubMed PMID: 18300940.

16. Al-Hendy A, Moshynska O, Saxena A, Feyles V. Association between mutations of the follicle-stimulating-hormone receptor and repeated twinning. *Lancet* (2000) 356(9233):914. Epub 2000/10/19. doi: 10.1016/s0140-6736(00)02687-8. PubMed PMID: 11036902.

17. Greb RR, Grieshaber K, Gromoll J, Sonntag B, Nieschlag E, Kiesel L, et al. A common single nucleotide polymorphism in exon 10 of the human follicle stimulating hormone receptor is a major determinant of length and hormonal dynamics of the menstrual cycle. *The Journal of clinical endocrinology and metabolism* (2005) 90(8):4866-72. Epub 2005/05/12. doi: 10.1210/jc.2004-2268. PubMed PMID: 15886248.
